# Epidural Gas Accumulation in Connection with Canine Degenerative Lumbosacral Disease

**DOI:** 10.3389/fvets.2017.00055

**Published:** 2017-04-18

**Authors:** Ditte Skytte, Hugo Schmökel

**Affiliations:** ^1^Ryggcenter, Specialistdjursjukhuset Strömsholm, Strömsholm, Sweden

**Keywords:** canine, lumbosacral degeneration, epidural gas accumulation, pneumorrhachis, disc protrusion

## Abstract

Three dogs were presented with lumbosacral hyperesthesia. Computerized tomography scans were performed in all the cases, and magnetic resonance imaging was also performed in cases 1 and 3. There was intervertebral disc (IVD) protrusion causing nerve root compression and epidural gas accumulation in all the three cases. The gas-filled cystic structures in cases 1 and 3 were within the spinal canal; in case 2, the gas was within the disc protrusion. The IVD vacuum phenomenon is relatively common in dogs, but the formation of an epidural gas accumulation in cases of a lumbar disc protrusion is rare. The clinical significance of these epidural gas accumulations is unknown. Two of the dogs were treated surgically, improved after surgery, and showed no signs of pain in the follow-up examinations.

## Introduction

Pneumorrhachis is a rare clinical diagnosis of gas within the spinal canal and was reported for the first time in human medicine in 1977 (1). After a trauma, gas can enter the spinal canal from a pneumothorax/pneumomediastinum or through open skull fractures (1–6). Epidural injections or surgical interventions in the head or spine can also lead to iatrogenic pneumorrhachis (7–12). Pneumorrhachis within the thoracolumbar spine causing spinal cord compression has been reported in two dogs (13, 14). Pneumorrhachis should be distinguished from disc-associated epidural gas accumulation, because in the former, the gas accumulating in the spinal canal originates from the intervertebral disc (IVD) vacuum phenomenon (VP) (14–16). Lumbar disc protrusion with epidural gas accumulation is reported in human medicine as a very rare form of the VP seen in association with a degenerating or infected IVD (17–22). The purpose of this study is to describe three cases of epidural gas accumulation with lumbar disc protrusion in connection with canine degenerative lumbosacral disease, a combination of conditions that has not previously been reported in dogs.

## Case Presentations

### Case 1

A 2-year-old intact male cane corso dog was presented for the evaluation and therapy of lumbosacral pain. The results of the general examination were unremarkable. During the orthopedic and neurologic examinations, a clear pain reaction was found during the palpation and extension of the lumbosacral junction. The computerized tomography (CT) and magnetic resonance imaging (MRI) scans showed reduced signal intensity and marked dorsal protrusion of the L7–S1 IVD together with a VP. A surgical dorsal decompression for removal of the protruded disc was performed, and the dog improved to a normal condition within 2 months of rehabilitation.

**Figure 1:**
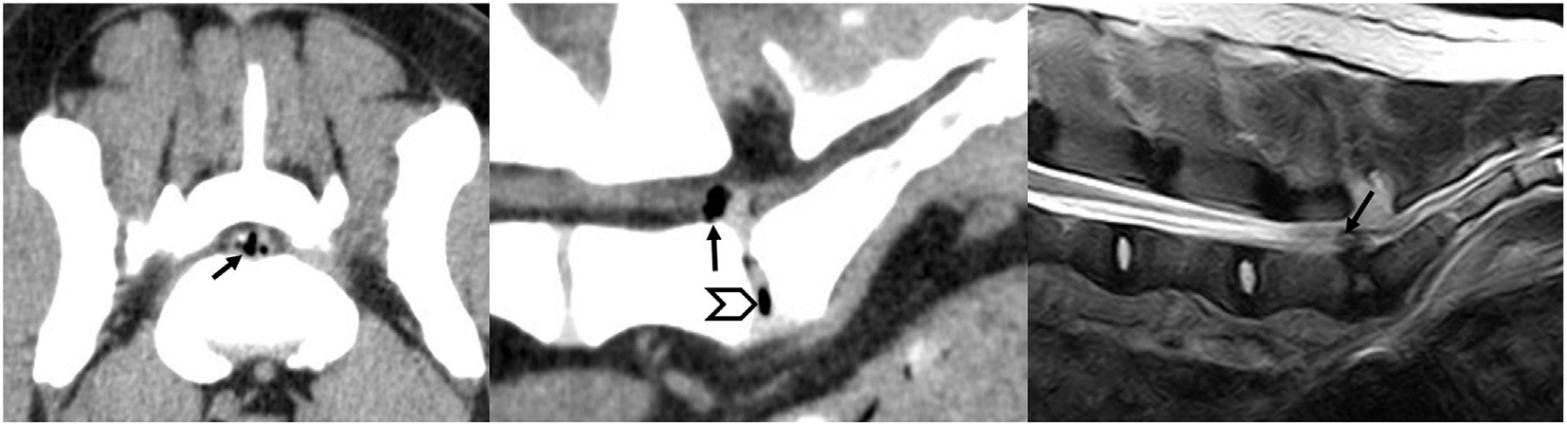
**Transverse computerized tomography (CT) and sagittal CT and magnetic resonance imaging images of case 1 showing the symptomatic compression of the cauda equina by a gas-containing disc protrusion (arrow) that appeared 3 years after the first surgical procedure done to remove the intervertebral disc protrusion. The vacuum phenomenon is visible on the sagittal CT image (arrow head)**.

Three years after that surgery, the dog was referred again because the clinical signs had recurred. Palpation of the lumbosacral junction caused pain, and the dog vocalized when standing up or climbing stairs. There were no neurological deficits. A new CT scan showed that a lumbar disc protrusion with an epidural gas accumulation had formed, compressing the cauda equina from the ventral side (Figure 1). Surgical exploration was recommended. The cauda equina was approached through the previously performed dorsal laminectomy of L7–S1. The gas-filled cystic structure was a smooth, soft tissue elevation from the vertebral floor and collapsed after an incision was made. The cauda equina was released from its adhesions to the spinal canal floor and IVD. The cystic wall and protruded IVD were removed with a partial discectomy. The closure with a fat graft was routine. The postoperative CT scan showed successful decompression, and the dog improved again and had no signs of lumbar pain in the 3-month follow-up examination.

### Case 2

**Figure 2:**
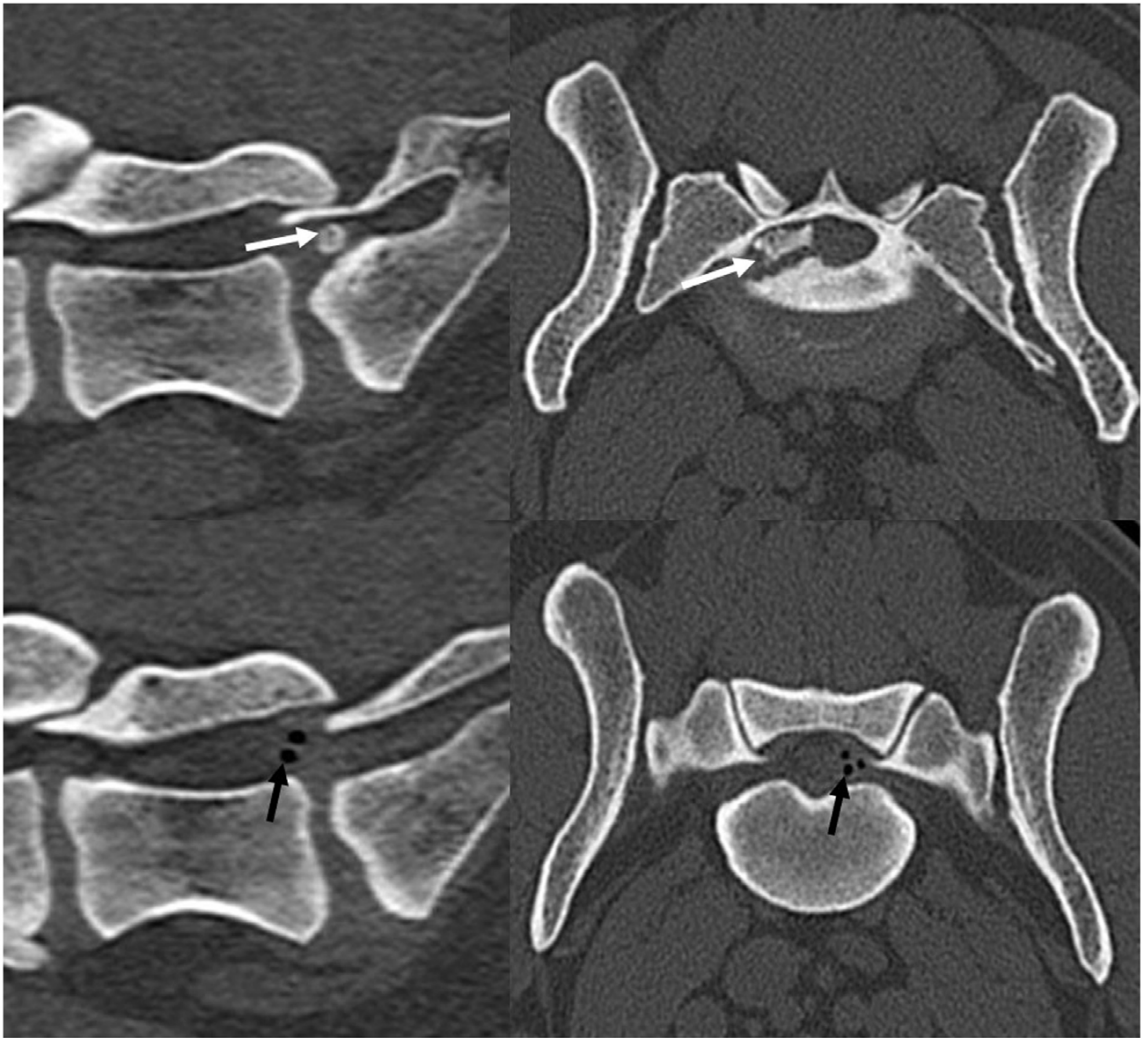
**A preoperative computerized tomography scan of case 2 showing the bony osteochondrosis dissecans fragment (white arrows) and the gas-containing disc protrusion (black arrows) affecting the cauda equina at the L7–S1 level**.

**Figure 3:**
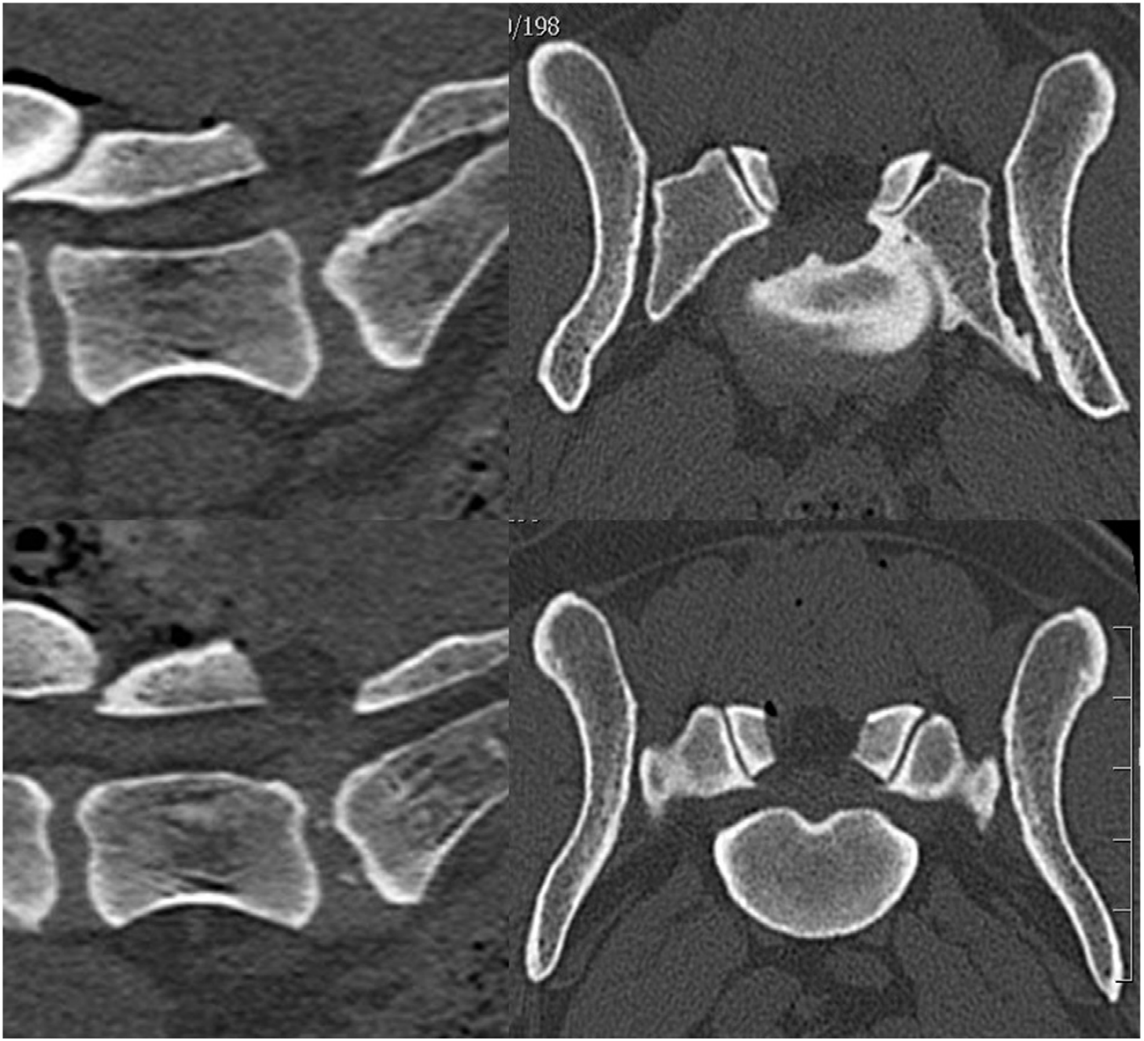
**A postoperative computerized tomography scan of case 2 showing that the bony osteochondrosis dissecans fragment and the gas-containing disc protrusion were removed**.

An 8-year-old intact female German shepherd dog was referred for further imaging and therapy for previously diagnosed pain in the lumbosacral area. The dog showed no neurological deficits of the pelvic limbs but did show signs of pain during jumping and climbing stairs. Conservative therapy with rest and non-steroidal anti-inflammatory drugs had not improved the clinical signs within 6 weeks. A CT scan revealed a sacral osteochondrosis dissecans (OCD) lesion with a large fragment in the vertebral canal on the left side caudal of the L7-S1 IVD. Cranial of the OCD fragment, an IVD protrusion with gas inclusion could be seen in the vertebral canal on the right (Figure 2). Because conservative treatment had already been unsuccessful, surgery was performed. The OCD fragment and the disc protrusion were removed *via* a dorsal laminectomy L7-S1 and a partial discectomy (Figure 3). After 6 weeks, the patient had improved and was moving upstairs without signs of pain; no pain was found by palpating the lumbosacral area. Three months after the surgery, the dog was walking and running normally.

### Case 3

**Figure 4:**
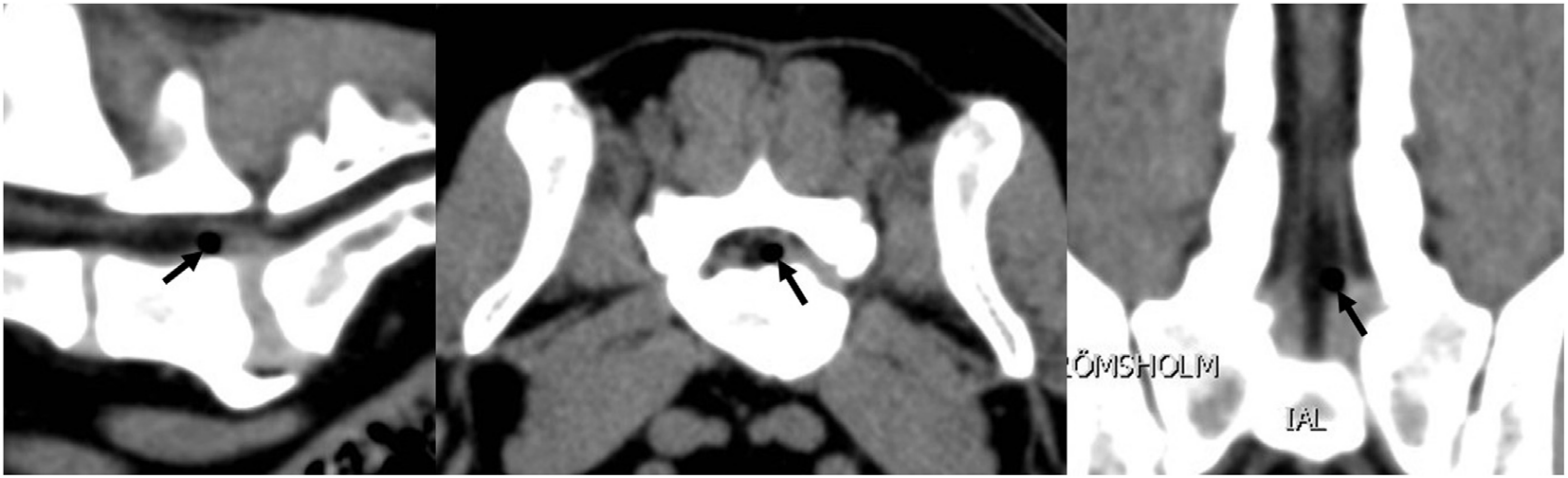
**A computerized tomography scan of case 3 showing the gas-containing disc protrusion (black arrows) affecting the cauda equina at the L7–S1 level**.

A 4-year-old castrated male French bulldog was referred with a history of progressing ataxia of the pelvic limbs. The blood values and results of the general examination were normal, but marked ambulatory ataxia of both pelvic limbs with reduced proprioception was recorded. The reflexes of the pelvic limbs were increased; the thoracic limbs were normal. In addition, strong pain was detected in the lumbosacral area. With the clinical localization of the neurological deficit causing pathology within the spinal cord segments Th3–L3 and the pain in the lumbosacral area detected during the clinical examination, CT and MRI scans of the thoracolumbar and lumbosacral spine were performed. In the area of Th6–Th7, malformations of the vertebrae with ventral spinal cord compression were detected together with a T2-hyperintense signal change within the spinal cord. These findings could explain the ataxia and proprioceptive deficits of the pelvic limbs. Furthermore, a small VP and other degenerative changes of the L7–S1 IVD were visible. The L7–S1 IVD was protruded, and a gas-filled cystic structure within the spinal canal had developed, leading to moderate ventral compression of the cauda equina (Figure 4). After discussing the findings regarding the lumbosacral spine, the dog’s owner decided not to decompress the cauda equina, because this condition was not the cause of the clinical signs. The ataxia and lumbosacral pain were treated conservatively with gabapentin and a non-steroidal anti-inflammatory medication in addition to rehabilitation. The dog was euthanized 5 months later because the ataxia of the pelvic limbs did progress to a non-ambulatory state.

## Discussion

Degenerative lumbosacral stenosis (DLSS) is an important cause of chronic pain in dogs (23). In this condition, numerous soft and bony tissue alterations of the spine exist, leading to the often exercise-dependent compression of the cauda equina, including the nerve roots in the spinal canal running over the lumbosacral IVD and/or exiting the lumbosacral foramina (24, 25). Most dogs with DLSS are affected by an IVD protrusion and proliferative soft tissue. An exact diagnosis of the individual’s compression pattern facilitates the successful planning of the surgical procedure. Dogs often respond well to dorsal decompression and partial discectomy, but malformations, instabilities, and bony proliferations or fragments have also been identified as factors causing clinically significant DLSS (25–27). The release of foraminal stenosis may be necessary and can be performed successfully (28, 29).

The VP, which refers to the presence of gas in the IVD, is a relatively common radiological finding in humans and dogs, especially from CT, with an incidence of 6% in canine vertebral CT scans (14, 16, 22, 30, 31). Although an intervertebral VP can indicate vertebral disc degeneration and herniation, it should not be considered a unique identifier for spinal compression. The localization for IVD surgery should be based on clinical signs, neurologic examinations, and CT and/or MRI findings showing compression of the spinal cord or nerve roots (31). The formation of an epidural gas accumulation is rare compared with the VP in an IVD. The VP is caused by the accumulation of gas in the IVD as it degenerates or the extrusion of nucleus pulposus material (Hansen type l herniation). It has also been associated with infection, invasive procedures, and trauma to the IVD (17, 22, 32, 33). The spaces created within the IVD fill up with nitrogen-containing gases that were originally dissolved in the extracellular fluid and diffuse into areas of subatmospheric pressure during the movement of the IVD (22, 34). If the annulus fibrosus ruptures, this air can be released within the spinal canal, creating a pneumorrhachis. The accumulation of this gas in the epidural space is an unusual cause of clinical symptoms. The gas can accumulate either within a herniated disc, like in case 2, or within a cystic structure, like in cases 1 and 3 (18–20, 34). The epidural gas accumulation can become surrounded by a fibrotic capsule and can grow with time (21). The ball-valve connection may cause an increase in the gas pressure inside the disc or cyst in the epidural space (35). The gas-filled cystic structures in the three patients in this study were part of the tissues compressing the cauda equina. The size of the gas-filled cystic structure was relatively large in case 1; in case 3, this structure occupied approximately 14% of the vertebral canal. The clinical significance of this epidural gas accumulation is unknown for both patients. In case 2, the gas inclusion was considered an incidental finding since the clinical signs were more likely caused by the OCD fragment and IVD protrusion. Should a gas-filled cystic structure grow over time, the clinical impact on the nervous tissue may increase.

Computerized tomography is the investigation of choice for diagnosis (14, 15, 34). CT scans not only show that the mass within the spinal canal is partially composed of gas but also provide useful information about the condition of the disc and the rest of the lumbar spine. Surgery is recommended and generally successful in human patients who fail to respond to conservative therapy for gas-containing lumbar disc herniations (18–21, 33–35). The two canine patients treated by surgical removal of the causes of the lumbosacral stenosis, including the epidural gas accumulation, improved after the procedure and rehabilitation.

## Concluding Remarks

The IVD VP is a sign of IVD degeneration and is relatively common in dogs, but the formation of a lumbar disc protrusion with an epidural gas accumulation is rare. This gas-filled cystic structure can be a part of tissues compressing the cauda equina; however, the clinical significance is unknown. In this study, the surgical treatment of the clinical DLSS by removing all the causes of the stenosis, including the epidural gas accumulation and disc protrusion, was successful in the two cases in which it was performed, although follow-up was limited.

## Ethics Statement

The study describes three cases of canine patients treated at our hospital following the best knowledge of the authors.

## Author Contributions

DS contributed to writing the manuscript and literature review. HS contributed to writing the manuscript and was the primary surgeon for the cases.

## Conflict of Interest Statement

The authors declare that the research was conducted in the absence of any commercial or financial relationships that could be construed as a potential conflict of interest.
